# Comment on October 2008 issue 4

**DOI:** 10.4103/0019-5413.40267

**Published:** 2008

**Authors:** TK Shanmugasundaram

Sir,

I wish to congratulate you for bringing a sea-change in the content and quality of the Indian Journal of Orthopaedics under your editorship.

Volume 41 Issue 4 contains a number of articles from authors from around the world, all describing various surgical techniques for the management of especially thoraco-lumbar vertebral injuries. There are a couple of articles on epidemiology. But there are none on conservative management and rehabilitation from the centres in India.

Most of the articles give cursory consideration to spinal cord injury (SCI). The improvement of at least one grade in Frankel's classification as described in most papers makes a mockery of improvement from Grade A to Grade B. It seems to be an exercise of fracture treatment and not holistic management of SCI patients.

Less than one per cent of the SCI patients reach the few tertiary hospitals with facilities and inclination to treat SCI patients. It is a poor man's injury. A good article describing the basic principles of care of SCI patients would go a long way in avoiding the innumerable complications these patients are heir to. In case no such paper was submitted, an invited paper would have done justice for the lacuna.

I wish my comments may be given due consideration for future thematic coverage by the Journal.

Further I wish to suggest starting a “Letters to the Editor” column which will give scope for the readers to express their views on the articles published in the Journal.

